# Notices for July

**Published:** 1873-07

**Authors:** 


### NOTICES FOR JULY.

### PUBLICATIONS OF J. B. FORD & CO., NEW YORK.

" *The Greatest of Living Preachers.*"

—Brit. Quar. Review.

BEECHER'S SERMONS.—Seventh Series, Sept., 1871—March, 1872. Eighth Series, March–September, 1872. By HENRY WARD BEECHER. 2 vols. 8vo., 450–500 pp. each. Cloth. Price, $2.50. Half morocco, $5 per vol.

These are the continuation of a series of weekly issues from Mr. T. J. Ellinwood's *verbatim* reports. Each volume contains twenty-six sermons, and the prayers before the sermons. These books are among the most interesting and instructive religious reading that can be procured. Mr. Beecher is not only an exponent of divine teaching, but he has also the gifts of human sympathy, keen observation and practical wisdom. His comparisons and illustrations are such as reach the understanding and touch the heart, and it does not need the famed oratory of the preacher to lend them power. Mr. Beecher's sermons are among the rare ones that retain their force, vigor and pathos when transferred to print. Also,

NEW LIFE AND NEW LANDS.—By GRACE GREENWOOD, being letters of travel to and from San Francisco by the Pacific rail, made in 1871. 413 pages. The subjects are Chicago, Colorado, Utah, Nevada, California, etc. It is a most instructive volume to all, especially those who contemplate a journey to the shores of the great Pacific sea; it is a book which can be read with amusement and solid profit before the journey and after the journey's end, pointing out what to see and how to see it. $1.50.

MOTHERLY TALKS WITH YOUNG HOUSEKEEPERS.—By Mrs. HENRY WARD BEECHER, with her portrait, 12mo., 492 pp., with 12 pages of contents, making the volume exceedingly convenient. It can be said with great truthfulness that a more valuable book for young housekeepers has never yet been printed; it is not confined to buying and curing meat and bread, but it extends to the important branch of inculcating great principles of action, the care and training of children, as to system, heedlessness, worth of a good wife, how to teach little boys to be useful, trust your children, stealing servants, parental example, procrastination, grumbling, and a multitude of other subjects, requiring several pages to enumerate. Such a book could not have been written by any other than a practically observant, thoughtful, and educated Christian woman; and, for the sake of husbands, wives, sons and daughters, there ought to be one in every family in the United States. Sent postpaid for two dollars. Also,

STAR PAPERS.—New edition, 447 pp., 12mo., $1.50. New edition, with additional articles selected from more recent writings, 1873. By HENRY WARD BEECHER.

THE GREAT EVENTS OF HISTORY, from the Creation of Man until the Present Time. 377 pp., 12mo.

With a very convenient index by WILLIAM FRANCIS COLLIER, LL.D., Trinity College, London; edited by an experienced American teacher. This is not only admirably adapted to schools and colleges, but would be of great advantage to every family of culture, and to every individual who loves useful reading, as it gives a connected view of the Christian era, and a succinct account of the nations of the earth, showing what they were and what they are. It may be read to advantage every year. The geographical appendix is of great value, giving in a few lines the description of the chief cities and nations of the globe. Every teacher ought to have it on his table.

VALUABLE READING for summer, when one does not want to think much, nor have the attention given to any one thing long, may be had by purchasing a bundle of old newspapers, containing one hundred, at any office, for twenty-five cents. Such a package, taken to the country or on shipboard, will be a source of entertainment, amusement, diversion and instruction, worth many times the cost of a book, and many times preferable. These twenty five cent bundles—or perhaps half a dollar now—are made up of the latest papers issued from every State and Territory in the Union, and treat shortly on every conceivable subject. Or, stop at a second-hand book store, and purchase, for eight or ten cents each, several old numbers of the *Atlantic Monthly*, *Harper's Magazine*, or *Scribner's*, and a wealth of amusement and instruction will be found in them which will surprise almost every one. The leisure of a country seat, or a sea voyage, will give an opportunity to read almost every article, and the wonder will immediately arise in the mind, how is it possible to get so much information for so small an amount of money? For those who prefer subjects of a more weighty character, and of deeper thought, a few numbers of *The Westminster Review*, *The London Quarterly*, or *Blackwood's Magazine* will answer an admirable purpose.

LAND SURVEYING, with Tables, by DAVID MURRAY, A. M., Ph. D., Professor of Mathematics in Rutgers College. Published by G. J. W. Scheimerhorn & Co., New York. 259 pp., 12mo.

Not only a text-book for schools but a surveyors' manual. It is a concise, practical treatise on land surveying, and is worthy of being made a standard publication.

BALDWIN is not only the prince of clothiers but he is the Napoleon of business men. One reason for his success is the promptitude and courtesy with which every person is waited upon who enters the establishment. In very many stores in New York the clerks actually wait until a customer comes to them at the rear of the building, or lazily saunter up to him, as if they would rather prefer that he would go away before they could reach him. Baldwin is a commanding general; we have seldom entered his establishment without seeing him in a position from which his eye could rest on every clerk, seeing for himself that all were at their posts, in diligent attention to their duties. Every man of the hundred knows that the eye of the master is on him, consequently the whole machinery of the place works quietly, admirably and successfully. Besides all this, Baldwin is a good citizen, his private character without a stain, and his commercial standing A No. 1. Going from clothing to milk, a friend once said that if he had to go over his life again he would embark in some business connected with a man's back or belly. The expression was not very elegant, but there was wisdom in the thought; for, said he, however dull the times, people will dress and eat; under all circumstances, and in the "hardest times," money will be forthcoming to purchase food and clothing. For many years S. W. Canfield, President of the Rockland County Milk Association, has been furnishing the families of New York with unadulterated milk of farm-house fed cows. As proof of its purity you have only to purchase some and let it stand, and the rich, thick, yellow cream begins at once to gather on the surface. We have taken his milk fifteen years, and know for ourselves whereof we affirm. Another of Baldwin's ways, which, if universally adopted, would sweep from the civilized world half its sorrows, is this: "I credit no man, nor will I ask any man to credit me." The result of this course is, Baldwin has no losses, and, consequently, can sell cheaper than those who do. Let the reader give this a serious thought.

Thesicki is a pamphlet of 80 pages, 8vo. Published by Dr. S. B. Collins, La Porte, Indiana. Sent, post-paid, for 50 cents. The amount of it is simply this, that the writer can eradicate the love of drink; can cure persons of drunkenness. It contains much useful and valuable information, and is well worth reading by all who are interested in drunkards.

Bits of Talk about Home Matters is a beautiful and entertaining volume for summer reading, from the house of Roberts Brothers, Boston, Mass. It is admirably instructive to parents and children.

The Year.—A beautiful volume of poetry for each month in the year, for Christmas and each of the seasons. By D. C. Colesworthy, and dedicated to John Neal. Lee & Shepard, Publishers, Boston; who also have issued "Little Grand Father." By Sophie May. Illustrated. Useful and instructive to all young readers, being one of Little Prudy's Flyaway Series.

New York State Inebriate Asylum, at Binghamton, being the Annual Report for 1872. D. G. Dodge, Superintendent. This is an interesting and very satisfactory document, which any one can get by sending 50 cents to F. L. Stone, Librarian ; it contains the rules, regulations, prices and success of the institution. The chaplain, Rev. Samuel W. Bird, while admitting the existence of an opinion that " inebriety is a disease requiring medical treatment, very properly states that the patient is himself responsible for it; that it is a moral disease, intensified by recklessness." Drunkenness is a crime, as much as gormandizing, gluttony and beastiality. The *roué* is a criminal. If it is intended to offer it, as an excuse for being a drunkard, that it is a disease, we object to it. If a man wants to avoid getting drunk he can do so. Might as well say that a person murderously inclined can't help it. They don't want to help it. Dr. Bird well calls it " reckless self-indulgence ;" they satify their gross appetites for the satisfaction it gives themselves, regardless of how it breaks the hearts of father and mother, and wife and children. There is no excuse, no apology for any human being who is a drunkard; it is no more or less than the quintessence of selfishness.

Civil Malpractice.—By M. A. McClelland, M. D. Published by W. B. Keon, Cooke & Co., Chicago; being a Report to the Military Tract Medical Society, January 14, 1873. Price, $2. No intelligent physician or lawyer can fail to read this Report with deep interest; important principles are discussed in reference to Medical Jurisprudence. All who have had any personal connection with suits for malpractice, or who have been witnesses in such cases, will be instructed by reading the Report.

Business Success.—As an evidence of what the sunshine is doing for trade, we are glad to mention the fact that Baldwin, the clothier, of Canal street and Broadway, received for clothing sold at retail, C. O. D., on Saturday, over six thousand dollars! But this is nothing for Baldwin. Great things are expected of him.

Going Abroad.—The fullest, briefest and best book for persons visiting Europe is Hurd & Houghton's "Satchel Guide," pocket size, flexible cover, 310 pages, besides 18 blank pages for keeping accounts and making notes. It is as succinct as possible as to routes, distances, modes of conveyances, prices, hotels, times of starting of steamships, rail cars and carriages. It tells you, in the fewest words possible, what to see, where to go, and how to get there, the value of the different coins, where to get your money changed, what to wear, how to travel, and a multitude of other things, which are seldom learned except by one's own experience. The information about how to make the voyage across the Atlantic is worth many times the cost to any one who crosses for the first time, and attends to its suggestions. Having gone abroad twice ourselves, the truthfulness of the statements made as to all points is pleasantly surprising. The publishers have done the general public a service in issuing such a book, and no traveller, who wishes to enjoy and to see a great deal at the least cost, and in the shortest time, can afford to be without "The Satchel Guide to Europe." Price, $2.

PILES.—William Wood & Co., Walker street, New York, have published an 8vo. monograb of 54 pages on The Physical Exploration of the Rectum, with an appendix on the Ligation of Hæmorrhoidal Tumors. By WILLIAM BODENHAMER, A. M., M. D. Illustrated by numerous drawings. This publication should find its way into the library of every educated physician in all schools, as Dr. B. is the most able and successful of living surgeons in the treatment of all maladies of the structures contagious to the locality of piles, etc., having for more than thirty years given his exclusive attention to their rectification. The book, in flexible cloth, is sent, post-paid, for one dollar.

We congratulate our old friend Jesse Larrabee on his accession to the Managerial Department of the New York and Boston Despatch Express. The Company have secured in him a thorough gentleman as well as an efficient business man. The Company's New York office is located on the corner of West Broadway and Thomas street, and, as their rates are 25 per cent. lower than any other line, are doing a very large business.

We would like to call the attention of our readers to the Turkish Baths of Dr. Chas. H. Shepard, at Nos. 81 and 83 Columbia Heights, Brooklyn, N. Y. For luxury and elegance they are unsurpassed in the country.

